# Remdesivir-Loaded Nanoliposomes Stabilized by Chitosan/Hyaluronic Acid Film with a Potential Application in the Treatment of Coronavirus Infection

**DOI:** 10.3390/neurolint15040083

**Published:** 2023-10-30

**Authors:** Viktoria Milkova, Neli Vilhelmova-Ilieva, Anna Gyurova, Kamelia Kamburova, Ivaylo Dimitrov, Elina Tsvetanova, Almira Georgieva, Milka Mileva

**Affiliations:** 1 Institute of Physical Chemistry ‘Acad. R. Kaischew’, 1113 Sofia, Bulgaria; 2 Stephan Angeloff Institute of Microbiology, Bulgarian Academy of Sciences, 1113 Sofia, Bulgaria; elinaroum@yahoo.com (E.T.); almirageorgieva@gmail.com (A.G.);; 3 Institute of Neurobiology, Bulgarian Academy of Sciences, 1113 Sofia, Bulgaria

**Keywords:** chitosan, remdesivir, aptamer, encapsulation, drug release, coronavirus HCoV-043

## Abstract

An object of the present study was the development of liposomes loaded with the medicine Veklury^®^ (remdesivir) stabilized by electrostatic adsorption of polysaccharide film formed from chitosans with different physicochemical characteristics and hyaluronic acid. The functionalization of the structures was achieved through the inclusion of an aptamer (oligonucleotide sequence) with specific affinity to the spike protein of the human coronavirus HCoV-OC43. The hydrodynamic size, electrokinetic potential and stability of the structures were evaluated at each step in the procedure. The encapsulation efficiency and loaded amount of remdesivir (99% and 299 µg/mL) were estimated by UV–vis spectroscopy. Our investigations showed manifestation of promising tendencies for prolonged periods of the drug release and increased effectiveness of its antiviral action. Among all studied versions of the delivery system, the most distinguished and suitable in a model coronavirus therapy are the liposomes formed from chitosan oligosaccharides. The cytotoxicity of the liposomes was determined against the HCT-8 cell line. A cytopathic effect inhibition test was used for the assessment of the antiviral activity of the compounds. The virucidal activity and the effect on the viral adsorption of the samples were reported by the end-point dilution method, and the alteration in viral titer was determined as Δlgs compared to untreated controls. The redox-modulating properties of the nanoparticles were studied in vitro in certain/several/a few chemical model systems. Our investigations showed a manifestation of promising tendencies for a prolonged effect of the drug release and increased effectiveness of its antiviral action.

## 1. Introduction

The outbreak of the world pandemic caused by the severe acute respiratory syndrome coronavirus 2 (SARS-CoV-2) directed profound research interest in exploring possibilities and discovering novel pathways to oppose the virus and/or alleviate the consequences in the human organism. Earlier studies on other members of the *Coronaviridae* family, such as the severe acute respiratory syndrome coronavirus (SARS-CoV) and the Middle East respiratory syndrome (MERS-CoV), provided valuable insight into the mechanism of infection because of their significant similarity to SARS-CoV-2 [1,2], which knowledge has been extensively developed during last years. 

Moreover, viral infections have a detrimental impact on neurological functions and cause neurological damage and an enhanced risk of severity and mortality. Additionally, patients suffering from rare diseases are extremely vulnerable to the detrimental impact of viral infections. 

Several reasons can be proposed to explain these observations: (i) most of the patients with neurogenerative brain disorders (NBDs) are old and have other comorbidities; (ii) patients with dementia are associated with the ApoE e4 genotype (associated with dementia and delirium) that can result in an increased risk of having severe coronavirus infection; (iii) the inflammatory state or viral infections in dementia patients may be caused by an increase in white blood cells count; (iv) people with NBDs, due to the nature of their cognitive decline, are unable to follow healthcare and preventive measures, therefore, making them at a higher risk of contracting an infection [3]. Therefore, these diseases can further be considered as an important factor and comorbidity in patients with viral infections requires an investigation into the possible mechanisms of neuro-invasion and management strategies.

The major role in the coronavirus invasion into the host cells is attributed to the spike S glycoprotein located on the spikes’ surface [4]. This makes the protein a key target for the diagnosis, treatment and vaccination of COVID-19 [5,6,7,8,9,10,11,12].

A possible strategy to inhibit the infection would be to target the S-protein before it connects to the receptor. The goal could be reached by using a competitive compound capable of disabling the contact region in protein by binding to it and, in such a way, preventing its interaction with the lipid membrane. To this purpose, antibodies or aptamers [13,14] have been applied, as the latter gain more and more research and clinical attention due to their low cost, longer life and stability, better immunogenic tolerability, possibilities to design and modify them, etc. [15,16].

The aim of the present high interdisciplinary study is to produce and characterize polysaccharide-stabilized liposomal carriers suitable for delivery of remdesivir with high loaded capacity.

Remdesivir, REM (also known as GS-5734 or medicine Veklury^®^), was chosen as an example medicine to address the infection, as it was found to act as an effective antiviral agent against a variety of RNA viruses, including Ebola, coronaviruses, etc. [17,18,19,20,21,22]. The drug is beneficial at later stages of the viral attack, in particular after fusion, endocytosis, translation and proteolysis inside the host cells, when it can integrate into the emerging viral RNA chains, interrupt transcription and finally hinder replication. According to in vitro experimental data, the inhibition of SARS-CoV-2 was above 90% at micromolar remdesivir concentrations (up to 100 μM), and its half-maximal effective concentration was estimated as low as 0.77 µM. Moreover, the cytotoxicity under the same conditions was relatively low, especially compared to other antiviral agents: the determined half cytotoxic concentration turned out to be greater than 100 μM.

Liposomal nanocarriers have been selected for the purposes of our research as a main transporting vessel, which fits the requirements for respiratory inhalation [23]. Known for their biocompatibility, immune tolerability, bio-absorbability by the cells due to the structural imitation of natural membranes, etc., liposomes represent a very advanced delivery container for a wide variety of drugs. These vesicles are composed of a phospholipid bilayer(s) surrounding the interior(s) of the aqueous solution, and their arrangement allows for loading hydrophobic molecules, incorporated into the hydrocarbon part of the bilayer, hydrophilic compounds, closed into the core, and surface active components, attached to the lipids [24,25,26,27]. After packing the drug, the liposomes could transport it in either a passive or active way. The first approach is usually limited to tumor treatment in relation to certain cancer characteristics and the preferential delivery to the affected tissues, while the second strategy leads to selective behavior of the carrier and affinity to certain types of cells [28,29]. Active targeting requires functionalization of the liposomal outer surface with specific binding ligands, such as aptamers [30,31].

In general, the liposomes form relatively unstable suspensions, tending to aggregate, and the system could be stabilized by the adsorption of polymers [32]. Therefore, the produced liposomes were stabilized by subsequent electrostatic adsorption of oppositely charged polysaccharides (chitosan and hyaluronic acid).

Chitosan, CS, is a family of cationic polysaccharides with versatile properties in the design of drug and gene delivery platforms with potential applications in medicine and biotechnologies. Their cationic properties make them beneficial for the formation of complexes with negatively charged molecules. A big diversity in chitosans is known, with different degrees of acetylation (DA) and molecular weight, which vary their physicochemical properties and, therefore, their utilization [33,34,35]. The DA is associated with the number of amino groups in the polysaccharide, which affects the charge and the molecule solubility and can shift the hydrophilic–hydrophobic balance. CS solubility is also pH sensitive, as it increases at low pH and reduces strongly in the pH range 6–6.5 (pKa 6.3).

Being an anionic polysaccharide, hyaluronic acid, HA, is able to adsorb electrostatically on the CS-coated liposome surface. HA has also been widely engaged in drug delivery formulations for the treatment of cancers, brain injuries, atherosclerosis, etc. and is prominent with extended periods of circulation and the specific targeting of CD44 cell receptors. HA molecular construction predicts the capacity for hydrogen bonding and the strong hydrophilic nature, which makes it an appropriate component for hydrogels in cosmetics and biomedicine [36,37,38,39]. In an aqueous environment, HA possesses properties of a weak acid, with pKa of carboxyl groups at ca. 3–4.

An aptamer (RNA sequence) with high affinity to the receptor-binding domain (RBD) of the spike protein of human coronavirus HCoV-43 is proposed to bind to the protein. However, we apply the aptamer not simply as an S-protein blockade but mainly as a guiding agent located on the outer surface of soft drug carriers (liposomes), leading them to the viral RBD. Our work suggests a combined double attack against the virus, including disabling the host-binding region of the S-protein and targeted drug delivery.

## 2. Materials and Methods

### 2.1. Materials

#### 2.1.1. Polysaccharides and Lipids

Chitosans, CS, purchased from Sigma Aldrich (Taufkirchen, Germany), were chosen for this study (product numbers 448869, 448877, 523682). The characteristics of polymers are presented in Table 1. The stock solutions were prepared with a concentration of 1 mg/mL in a solution of hydrochloric acid with pH 4.04. The solution of chitosan oligosaccharide, COS, was prepared in double distilled water. Before usage, the solutions were filtered through a 0.45 µm filter (Minisart^®^, Sartorius, Gottingen, Germany) to remove possible aggregates.

Samples of hyaluronic acid and sodium salt were also acquired from Sigma Aldrich (product numbers 40583 and 75044). The stock solutions (2 mg/mL) were prepared in double distilled water.

The stock solution of remdesivir (Veklury^®^, Gilead Science Inc. Ireland UC, Dublin, Ireland) was prepared in double distilled water, and the concentration of remdesivir in the stock solution was estimated to be 8.3 × 10^−3^ M. Betadex sulfobutyl ether sodium (SBECD), a product by Sigma Aldrich (product number PHR2923), was used in comparative experiments.

The phospholipid 1,2-dioleoyl-sn-glicero-3-phoshocholine (DOPC, chloroform solution, 25 mg/mL), a product by Avanti Polar Lipids Inc., was used for the production of unilamellar liposomes.

The aptamer selection was performed by using a web server called PRIdictor (Protein–RNA Interaction predictor) [40]. PRIdictor was used as a web-based application at http://bclab.inha.ac.kr/pridictor (accessed on 22 October 2023). The selected oligonucleotide sequence of aptamer (5′-AAA CAU UGC AC-3′) was synthesized from Biomers (Ulm, Germany). The sample was dissolved in double distilled water, and the concentration of the stock solution was 60.76 μM.

#### 2.1.2. Viruses

The human Coronavirus OC43 (HCoV-OC43) (ATCC: VR-1558) strain was propagated in human colon carcinoma cell line HCT-8 in RPMI 1640 medium supplemented with 2% horse serum, 100 U/mL penicillin, and 100 μg/mL streptomycin. Cells were lysed 5 days after infection by 2 freeze and thaw cycles, and the virus was titrated according to the Reed and Muench formula. Virus and mock aliquots were stored at −80 °C.

#### 2.1.3. Cytotoxicity Assay

A confluent monolayer cell culture in 96-well plates (Costar^®^, Corning Inc., Kennebunk, ME, USA) was treated with 0.1 mL of well-containing maintenance medium that did not sustain/or sustained decreasing concentrations of test substances. The 50% cytotoxic concentration (CC_50_) was defined as the concentration of the material that reduces cell viability by 50% compared to untreated controls. Each sample was tested in triplicate with four wells for cell culture on a test sample. The maximum tolerable concentration (MTC) is also determined, which is the concentration at which the compounds do not affect the cell monolayer in the sample, and it looks like the cells in the control (untreated with compounds).

### 2.2. Methods

#### 2.2.1. Liposome Preparation

The liposomes were prepared using the thin-film hydration method. An appropriate volume (200 µL) from the solution of lipid in chloroform (25 mg/mL) was dried under a stream of nitrogen by rotating the flask to form a thin lipid film on its wall. The lipid was re-hydrated in a solution of HCl (pH ~ 4.04) or a solution of REM (500 µM or 9 mg/mL, Veklury^®^) to a final lipid concentration of 2.5 mg/mL. The solution was frozen with liquid nitrogen, and 4 freezing/heating cycles were performed. The stock solution of liposomes was sonicated in an ultrasonic bath for 15 min. In order to prevent a possible aggregation during the subsequent steps in the experimental procedure, the concentration of the dispersion was adjusted to 0.02 mg/mL in a solution of HCl (pH ~ 4.04) followed by extrusion through a 0.20 µm filter (Minisart^®^, Sartorius). (The estimated concentration of liposomes in the samples was 3.5 × 10^14^ liposomes/mL).

**Figure 1:**
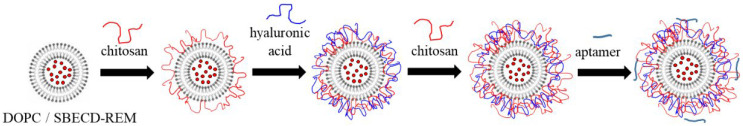
Procedure for preparation of polymer-stabilized liposomes for encapsulation of remdesivir.

The steps in the experimental procedure for the formation of the structures are presented in Figure 1. In order to improve the stability of the loaded liposomes, the first chitosan layer was adsorbed on the surface. It was formed by adding a diluted dispersion of liposomes (5 mL) to the solution of positively charged chitosan (0.5 mL, 1 mg/mL) and stirring for 20 min. This procedure was repeated by insertion of the chitosan-coated liposomes into the solution (4 mL) of negatively charged HA (1 mL, 2 mg/mL). The formation of the third polymer layer was performed in the same manner—dispersion of liposomes (4 mL) was added to the solution of chitosan (0.4 mL, 1 mg/mL). The final concentrations of chitosan and hyaluronic acid in the dispersion were 0.1 mg/mL and 0.5 mg/mL, respectively.

In order to ensure control over the concentration of the components in the suspension, the excess non-adsorbed polysaccharide molecules were not removed from the dispersion before each subsequent adsorption step (in correspondence to the electrokinetic measurements, the concentrations of polymers were high enough to ensure an overcompensation of the liposome net charge and re-stabilization of the suspension.)

In the last deposition step, an aptamer molecule (5′-AAA CAU UGC AC-3′) with a concentration of 5 μM was adsorbed by the chitosan-stabilized liposomes. The dispersion (2 mL) and the aptamer solution (0.164 mL) were mixed by using Vortex for a minute, and then the produced final sample was placed to rest without stirring for 20 min at room temperature (24 °C) (an approximate estimation shows that the number of aptamer molecules per liposome is ca. 9 × 10^3^ and chitosan-stabilized liposome/aptamer mass ratio is ca. 7.4).

#### 2.2.2. Determination of the Amount of Encapsulated Remdesivir

The concentration of the drug loaded into the liposomes was determined by the difference between the initial concentration of the compound added during the preparation of the liposomes and the concentration in the supernatant after centrifugation of the stock dispersion. The centrifugation was performed at 15,000 rpm (21,382 *g*, 15 °C) for 90 min, and the concentration of free SBECD–REM complexes was estimated by monitoring with a T60 UV–Visible Spectrophotometer (PG Instruments Limited, Leicestershire, UK). Remdesivir was registered at a wavelength of 242 nm, corresponding to the maximum absorbance peak of the SBECD–REM complexes, and the amount of free drug in the solution was calculated using a calibration curve. The encapsulation efficiency (EE%) was calculated by using the following relation.

EE% = (C_total_ − C_free_)/C_total_ × 100 (1)

where C_total_ is the initial concentration of the REM added to the dispersion (301 µg/mL) and C_free_ is the estimated concentration of the compound in the supernatant after the encapsulation.

The drug loading capacity (in %) was calculated with the following equation

LC% = (m_encapsulated_/m_capsules_) × 100 (2)

where m_encapsulated_ is the amount of drug successfully loaded into the carriers (in µg/mL), m_capsules_ is the number of capsules (490 µg/mL).

The correction in the encapsulation efficiency was conducted after the adsorption of the first chitosan layer. The diluted dispersion polymer-stabilized liposomes were centrifuged at the same conditions and the concentration of free SBECD–REM complexes was estimated.

#### 2.2.3. Determination of the Electrokinetic Charge and the Size of the Composite Liposomes

The size (hydrodynamic diameter) of the produced liposomes was evaluated after each step in the experimental procedure by using dynamic light scattering with non-invasive backscattering (DLS–NIBS, measuring angle 173°). The width of each polysaccharide layer was determined from the comparison of the liposome size before and after adsorption. For estimation of the film thickness, it was assumed that the regular polymer adsorption is achieved on the surface and the spherical shape of the formulation does not change.

The ζ-potential was determined by mixed-mode measurement phase-analysis light scattering. Dynamic light scattering measurements were carried out using Zatasizer Pro (Malvern Panalytical Ltd., Malvern, UK). All measurements were performed at 24.0 ± 0.1 °C. After five measurements, the average value was taken as the electrokinetic potential and size of the capsules.

#### 2.2.4. Drug Release

The release of REM was achieved by using a dialysis method. Briefly, after adsorption of the aptamer aliquot (1 mL) from the dispersion, it was added into a dialysis tube (D-Tube™ Dialyser Midi, MWCO 3.5 kDa, Sigma Aldrich) and incubated with phosphate buffer pH 7.00 (25 mL) (Chem-Lab NV, Zedelgem, Belgium) at room temperature (24 °C) with a stirring speed ca. 50 rpm. Aliquots (2 mL) were drawn at predetermined time points from the medium, and the medium was immediately replenished with fresh buffer. The concentration of free drug in the samples is estimated by UV–vis spectroscopy by using appropriate calibration curves.

#### 2.2.5. Host Cell Culture

Human colon carcinoma (HCT-8) cells were purchased from the American Type Culture Collection (ATCC). Permanent HCT-8 (HRT-18) (ATCC-CCL-244, LGC Standards) were maintained at 37 °C and 5% CO_2_ using sterile RPMI 1640 (Roswell Park Memorial Institute Medium, ATCC-30-2001) supplemented with 0.3 mg/mL L-glutamine (Sigma-Aldrich, Darmstadt, Germany), 10% horse serum (ATCC-30-2021), 100 UI penicillin and 0.1 mg streptomycin/mL (both purchased from Sigma-Aldrich).

#### 2.2.6. Antiviral Activity Assay

The cytopathic effect (CPE) inhibition test was used for assessment of the antiviral activity of the tested samples. A confluent cell monolayer in a 96-well plate was infected with 100 cell culture infectious dose 50% (CCID_50_) in 0.1 mL (coronavirus OC43 strain). After 120 min of virus adsorption, the tested sample was added in various concentrations, and the cells were incubated for 5 days at 33 °C and 5% CO_2_. The cytopathic effect was determined using a neutral red dye uptake assay, and the percentage of CPE inhibition for each concentration of the sample was calculated.

#### 2.2.7. Virucidal Assay

Samples of 1 mL containing HCoV (CCID_50_) and samples in their maximal tolerable concentration (MTC) were contacted in a 1:1 ratio and subsequently stored at room temperature for different time intervals (15, 30, 60, 90 and 120 min). Then, the residual infectious virus content in each sample was determined by the end-point dilution method of Reed and Muench [41] and the reductions in viral titer Δlgs were evaluated as compared to the untreated controls.

#### 2.2.8. Effect on the Viral Adsorption

Twenty-four-well plates containing HCT-8 cell monolayers were pre-cooled to 4 °C and inoculated with CCID_50_ of HCoV. In parallel, they were treated with tested samples at their MTC and incubated at 4 °C to give time for virus adsorption. At various time intervals (15, 30, 45 and 60 min), the cells were washed with PBS to remove both the compound and the unattached virus; then, the cells were covered with a support medium and incubated at 37 °C and 5% CO_2_ for 24 h. After freezing and thawing three times, the infectious viral titer of each sample was determined by the final dilution method. Δlgs were estimated compared to the viral control (untreated with the compounds). Each sample was prepared in four replicates.

#### 2.2.9. Statistical Analysis

Data on cytotoxicity and antiviral effects were analyzed statistically. The values of CC_50_ and IC_50_ were presented as means ± SD.

#### 2.2.10. Transmission Electron Microscopy (TEM)

The produced liposomes were visualized by TEM. The samples were prepared by dropping and drying a drop of suspension of capsules on formvar-covered TEM grids. The images were captured using a High-Resolution Scanning Transmission electron microscope HR STEM JEOL JEM 2100 for investigations of surface morphology.

#### 2.2.11. Ferric Reducing Antioxidant Power (FRAP)

The FRAP assay was performed according to Benzie and Strain [42] with some modifications using the following solutions: (1) 30 mM acetate buffer at pH 3.6; (2) 1 mM TPTZ (2,4,6-Tri(2-pyridyl)-s-triazine) in 40 mM HCl; (3) 1.5 mM FeCl_3_. Thus, the prepared solutions were mixed in the following ratio: 10 parts (1): 1 part (2): 20 parts (3). To a 50 mL sample in a disposable test tube, 1.5 mL of reaction mixture was added: blank–reaction mixture and 50 mL of deionized H_2_O. The principle of the method includes the reduction of a Fe (III) ion if the sample contains a reductant (antioxidant) to Fe (II) at low pH. The colorless Fe (III)–TPTZ complex is transformed into the blue Fe (II)–TPZ complex after incubation for 4 min at 37 °C. Absorption was measured at 593 nm. The results were expressed as µmol Trolox equivalents per g of dry substances.

#### 2.2.12. Cupric-Reducing Antioxidant Capacity

The Cupric-Reducing Antioxidant Capacity (CUPRAC) of the samples was calculated according to Apak et al. [43] and presented as µmol Trolox equivalent/g of substances (TE/g).

#### 2.2.13. Iron-Chelating Power

The reaction of iron (II) ions with ferrozine produces a pink complex with a maximum absorption wavelength of 562 nm. The addition of a sample containing a chelating agent lowers the observed absorbance. The experiments were carried out according to the following procedure: 0.2 mL of sample solution was mixed with 0.74 mL of 0.1 M sodium/acetate buffer (pH 5.23) and 0.02 mL of 2 mM FeSO_4_ in 0.2 M HCl. After 10–15 s, 0.04 mL of 5 mM ferrozine was added. After 10 min of staying in the dark, the absorbance was measured. The formula for determining the Fe (II) chelating capability of the tested material is:

(3) $$Activity\%=\frac{Ac-As}{Ac}\times 100$$

where Ac is the absorbance of the blank probe, containing all additional compounds, excluding the sample −200 µL sodium/acetate buffer. As is the absorbance of the sample solution [44]. The Fe (II) chelating capability is expressed as mM EDTA equivalent per 1 g of substances.

#### 2.2.14. DPPH Assay

DPPH (2,2′-diphenyl-1-picrylhydrazyl radical) analysis was conducted by the method described by Brand-Williams et al. [45]. First, 500 μL of the test solutions were added to 500 μL of a freshly prepared solution of 0.1 mM DPPH in methanol and incubated in the dark for 30 min. Then, the absorbance at 517 nm (A517) was measured against a mixture of DPPH solution and methanol (1:1) as a control. All data are presented as % scavenging effect of the activities versus control. The scavenging activity percentage (AA%) was determined as follows:

(4) $$AA\%=100-\frac{\left(Abs(sample)-Abs(blank)\times 100\right)}{Abs(control)}$$

## 3. Results

### 3.1. Characterization of the Drug-Loaded Liposomes

According to the experimental results, the size (diameter) and electrokinetic ζ-potential of the three types of blank liposome samples (unloaded liposomes and liposomes loaded with SBECD or SBECD–REM complexes) have almost the same values (size is ca. 38 nm and ζ-potential is ca. −55 mV). This outcome hints that the dimensions and the electric properties of the liposomes, which are not coated by polysaccharides, are defined mainly by the lipid characteristics, as well as by the specific preparation procedure of the formulations, and the loaded compound does not contribute essentially.

**Figure 2:**
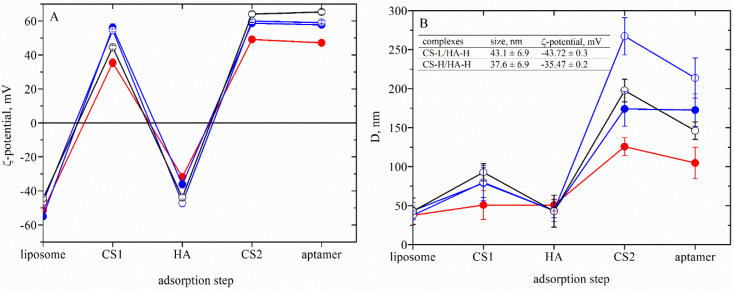
Characterization of the produced liposomes: dependences of ζ-potential (**A**) and hydrodynamic size (diameter), D, (**B**) of the composite liposomes after each adsorption step produced from COS/HA-L (●), CS-L/HA-L (●), CS-L/HA-H (○) and CS-H/HA-H (○). Inset: The characterization of complexes of CS-L/HA-L and CS-H/HA-L. Concentrations of the polysaccharides are CS (0.1 mg/mL) and HA (0.5 mg/mL).

Figure 2A shows the variation in the ζ-potential of drug-loaded liposomes after the deposition of each layer. The data indicate an overcompensation of the electrokinetic charge after the sequential deposition of oppositely charged chitosan and hyaluronic acid.

The evolution in the hydrodynamic size of the liposomes stabilized by polymer film is presented in Figure 2B. Depending on the balance of the charge density between the chitosan and the lipid membrane, the adsorption of polymer molecules on the zwitterion lipid layer could be governed by electrostatic interactions or the formation of hydrogen bonds between polysaccharide monomers and phospholipid head groups [46,47,48,49,50,51]. Because of the registered high electrokinetic potential of the liposomes at the present experimental conditions, we assumed that electrostatic adsorption occurs on the surface. The estimated values of the thickness of the first polymer layer (CS1) indicate a correlation with the molecular weight of chitosan (COS—13 nm, CS-L—35 nm and CS-H—50 nm).

The subsequent deposition of oppositely charged hyaluronic acid results in a significant decrease in the dimensions of liposomes. In spite of the registered overcompensation of the ζ-potential, the reduction of the hydrodynamic diameter hints at notable desorption of the polysaccharide film.

One possible explanation of the observed outcome is the variation in the adsorption behavior of the chitosan samples due to the significant difference between the polymer chain length and liposome size. According to the calculated length of the polysaccharide chains in Table 1, the chain dimensions of the chitosan molecules are 17 nm (for COS), 453 nm (CS-L) and 944 nm (CH-M), and the diameter of the liposomes is ca. 38 nm. Many theoretical and experimental studies on the polyelectrolyte adsorption on particles have shown that the curvature of the surface is a key parameter for the formation of a stable complex [52,53].

On the basis of the theoretical predictions, the interaction of a liposome with a polyelectrolyte molecule, both having similar sizes, results in the formation of a stable complex with a high fraction of monomers in trains (denser layer) and low fractions of monomers in loops and tails. Meanwhile, the deposition of polyelectrolytes with a high degree of polymerization on a small curved area does not allow the molecule to spread in the same way as on a flat surface, which is supposed to cause the formation of long loops and tails from the surface. In the limit of very small liposomes, the situation can be modeled as the adsorption of liposomes on polyelectrolyte molecules.

The addition of the oppositely charged molecules of hyaluronic acid is a critical step for the stability of the formed liposome/chitosan complex. It is reasonable to assume that the attractive electrostatic interaction between chitosan and hyaluronic acid monomers will be more favorable compared to the connection of chitosan with the liposome, which results in desorption of the complex CS/HA in the solution (both of the polysaccharides are fully charged at the present experimental conditions and their polymer lengths are compatible, Table 1). Moreover, a negative effect of the uncovered zwitterion surface of liposomes on the adsorption of HA can be expected because of the appearance of repulsive electrostatic forces between the same-sign charges in the lipid headgroups and HA molecules.

**Figure 3:**
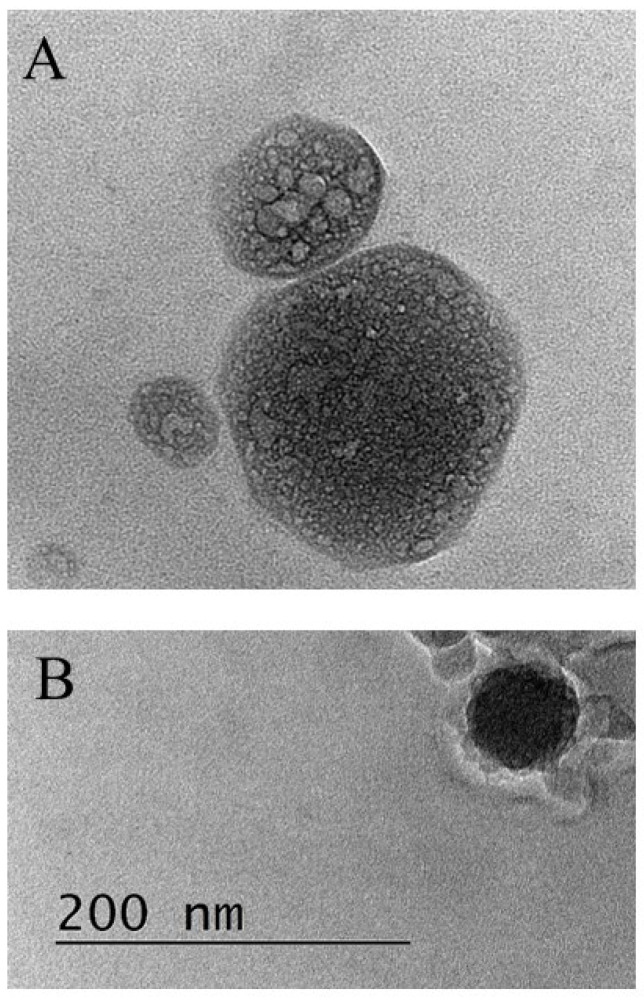
SEM images of unloaded liposomes without polyelectrolyte film (**A**) and drug-loaded liposomes covered by film from COS/HA-L/COS/aptamer (**B**). The dimension bar is corresponding to both of the micrographs.

During the addition of chitosan in the next step in the procedure (CS2), because of the excess positive charge, it is supposed that the previously desorbed CS/HA complexes in the solution can adsorb back on the coated liposome surface (Figure 3). Moreover, the significant increase in the hydrodynamic size of the liposomes could also be a result of the aggregation in the dispersion. Further investigation of the interlinkage of chitosan and hyaluronic acid with the same concentrations indicates the formation of stable complexes having a size and ζ-potential very close to the registered ones from the dispersion of liposome after the addition of HA (Inset in Figure 2B).

**Figure 4:**
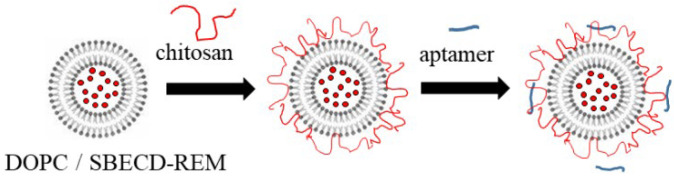
Simplified procedure for formation of chitosan-stabilized liposomes for encapsulation of remdesivir.

**Figure 5:**
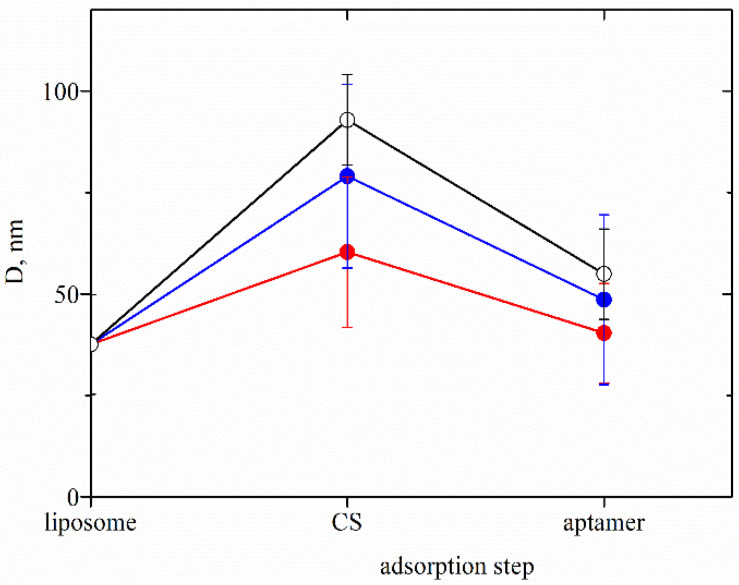
Evolution in the film thickness of liposomes stabilized by film from chitosan with different physicochemical characteristics and aptamer: COS (●), CS-L (●), CS-H (○).

According to the presented results, the dispersion of liposomes coated with COS and low molecular hyaluronic acid is a more stable and controlled system compared to CS-L/HA-L, CS-L/HA-H and CS–H/HA-H, where desorption occurs as already discussed. (Figure 2B). That is why the sample COS/HA-L is used further for microbiology and oxidative stress studies against human coronavirus strain OC43 and HCT-8 cell line. Based on the presented above, a simplified procedure for the formation of more stable formulations was proposed, suitable for model inhalation administration (Figure 4). The produced liposomes are stabilized only with the adsorption of a single chitosan layer. The evolution in film thickness of the system is presented in Figure 5.

The adsorption of a very low concentration of aptamer in the last step results in a slight decrease in the electrokinetic potential and the size of the liposomes produced following both procedures (Figure 1 and Figure 4). Because of the strong electrostatic interactions between oppositely charged chitosan monomers and aptamer molecules, the film is shrinking, which corresponds to the reduced film thickness (An approximate estimation shows that if it is assumed that the molecules are deposited only on the film surface, the number of aptamer molecules per liposome is ca. 2 × 10^4^).

### 3.2. Loaded Amount of Remdesivir

The amount of remdesivir entrapped into the liposomes during their formation (ca. 295 µg/mL) is estimated by the difference between the initial concentration of the compound added during the preparation of the liposomes and the concentration in the supernatant after centrifugation of the stock dispersion. The calculated encapsulation efficiency is ca. 98%.

**Figure 6:**
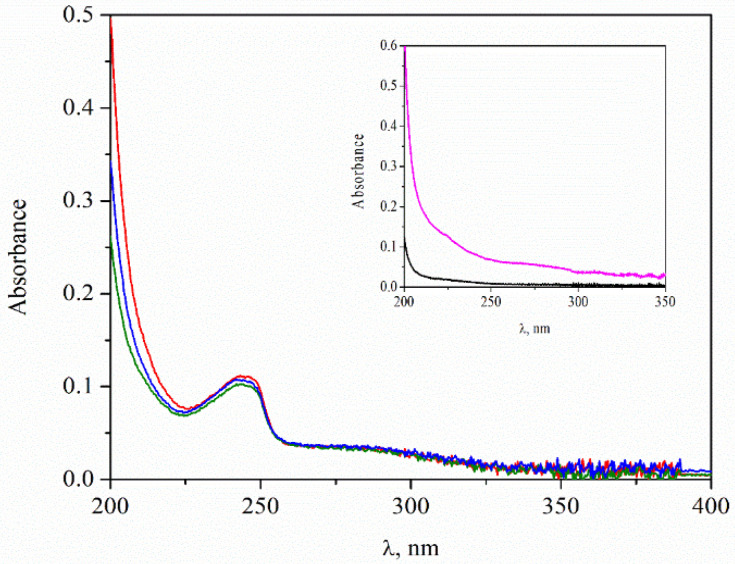
Spectrophotometric results: absorbance from the supernatant of dispersion of liposomes after adsorption of chitosan: COS (red), CS-L (blue) and CS-H (green). The inset: spectra of SBECD: 10 mg/mL (black) and 50 mg/mL (magenta).

In accordance with the proposed procedures, in the first step, chitosan molecules are adsorbed on the liposomes in order to improve their stability. Since the free SBECD–REM complexes in the dispersion (left after the encapsulation) are negatively charged, we supposed additional adsorption of the drug-containing complexes into the polysaccharide layer because of the electrostatic interactions with the positively charged chitosan monomers. That is why the loaded amount of remdesivir is re-estimated (Table 2). For this purpose, the dispersion of liposomes stabilized by a single chitosan layer is centrifuged, and the amount of free drug in the supernatant is determined by using a calibration curve (Figure 6). The estimation indicates that the EE% almost does not depend on the physicochemical characteristics of chitosan.

In the present study, remdesivir is loaded onto the liposomes not as a pure compound but as a complex of SBECD–REM. Therefore, the encapsulation efficiency and the release of the drug are estimated by using calibration curves of SBECD–REM. That is why, to clarify the effect of SBECD on the results for EE% of remdesivir, liposomes loaded solely with SBECD were prepared and studied. The values of their size and electrokinetic potential were found to be very close to those of the liposomes loaded with Veklury^®^, which confirms that the properties of the SBECD are essential for the encapsulation of the drug.

Otherwise, according to the spectrophotometry measurements, SBECD has no characteristic peak at the wavelength range where the drug was detected (inset in Figure 6). Therefore, the registered peaks and the estimated amount of the drug correspond to remdesivir only.

It was found in the literature that only a few studies addressed the encapsulation of pure remdesivir, but there are no investigations with Veklury^®^. Table 3 presents the comparison of the physicochemical characteristics and encapsulation efficiency of the drug-loaded structures.

A comparison between the experimental results (presented in Table 1 and Table 2, Figure 2) and the reported data indicates that using the proposed procedures achieved extremely high encapsulation efficiency in correlation with higher stability (high values of ζ-potential, respectively) of the structures, higher loaded amount of drug and lower liposomal size.

### 3.3. Release of Remdesivir from the Produced Liposomal Structures

**Figure 7:**
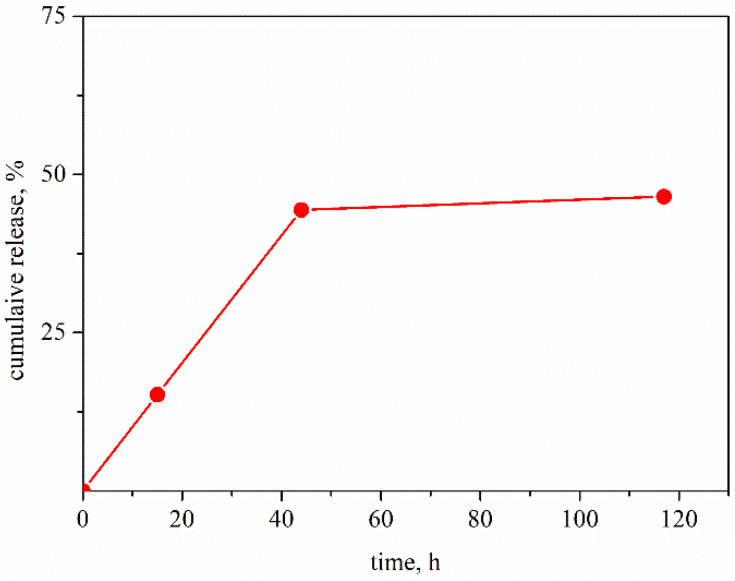
Estimated release amount of remdesivir from liposomes stabilized by chitosan oligosaccharide, COS and aptamer in phosphate buffer pH 7, 37 °C.

The concentration of free drug in the samples is estimated by UV–vis spectroscopy by using appropriate calibration curves. The experimental results indicate that the released amount of the drug almost does not depend on the chitosan sample and achieved almost 50% free REM in dispersion after 120 h (Figure 7).

### 3.4. Cytotoxicity Assay

The cytotoxicity of pure polymer solutions and the samples of produced liposomes are tested against the HCT-8 cell line. In our previous study, it was obtained that the pure solutions of CS-L and CS-M have very low antiviral activity against the HCT-8 cell line [59]. Therefore, in the present microbiology studies, only the solution of COS and the samples of liposomes stabilized with this polymer are used.

All experimental samples showed low toxicity (Table 4). According to the experimental results, the MTC of pure polymer is significantly higher compared to the liposomes. Moreover, the MTC does not depend on the number or the structure of polysaccharide film on the liposome surface.

According to the presented results, in spite of the high encapsulated amount of the drug, the liposomes manifest negligible antiviral activity towards HCoV-OC43. We supposed that the observation results from the low sensitivity of the experiment.

For comparison, previously, it was obtained that the MTC of the pure medicine Veklury^®^ against the HCT-8 cell line is ca. 1000 µg/mL. In the presence of human coronavirus HCoV-OC43, the estimated experimental value of IC_50_ was ca. 12.5 [59]. In the present study, the MTC of the liposome samples corresponds to encapsulated ca. 30 µg/m REM and can expect almost two orders lower values of IC_50_. The expected released amount of free drug in the cell medium during the experiments is lower than 50%. Therefore, it can be supposed that the effect of the presence of the drug is very low, and the values of IC_50_ can not be determined correctly.

### 3.5. Influence on the Replication Cycle of Human Coronavirus Strain OC43

The polymers and liposomes were investigated for their effect on the intracellular replication cycle of the HCoV-OC43. The polymers administered individually showed inhibition of viral replication with selectivity index SI = 52.9 (for chitosan) and SI > 8.0 (for hyaluronic acid), with COS exhibiting higher inhibition potential. The samples of liposomes did not show any activity.

### 3.6. Effect on Extracellular Virions of Human Coronavirus Strain OC43

Table 5 shows the results from the effect of the presence of polymers or liposomes on the extracellular virions of HCoV-OC43. The pure solutions of COS and HA showed an effect (most likely non-specific) on the extracellular virions. Even in the first investigated time interval (15 min), HA showed a significant inhibition of viral particles, reducing the viral titer by Δlg = 2.0, whereas chitosan exerted a weak inhibition of viral particles. The effect is time-dependent, and as the exposure time progresses, the inhibitory effect also increases—30 min after the exposure; the effect is already significant with chitosan, which leads to a decrease in the viral titer with Δlg = 2.0. By the last observed time interval (120 min), the decrease in viral titer reached Δlg = 2.66 for COS and Δlg = 3.0 (HA). It is assumed that the electrostatic interaction between the polycationic positive charge of chitosan and the negatively charged surface of the virus takes place. This binding can inhibit the infectivity of the virus and/or directly kill the virus by disrupting its protective membrane.

Taking into account the specific mechanism of action of REM on the virus, in particular its involvement in the inhibition of viral RNA transcription and replication inside the host cells, there is no surprise that the drug has no measurable effect on the extracellular virions.

### 3.7. Effect on Viral Adsorption

Similar results are observed in the influence of the tested samples on the stage of adsorption of the virus to sensitive cells. Only pure COS and HA exerted an inhibitory effect on the adsorption step of HCoV-OC43 on HCT-8 cells: at 15 min post-exposure, inhibition with HA was weak Δlg = 1.5, while chitosan more significantly affected this stage with Δlg = 1.75. The effect is also time-dependent and increases up to 120 min with Δlg = 3.25 for chitosan and with Δlg = 2.5 (HA) (Table 6).

Although chitosan is able to inhibit viral adsorption to host cells, the chitosan-coated liposomes have negligible contribution to the process. We assume the result could be related to the different effective surface areas of contact in the case of free polymer molecules and after they form a film over a small, approximately spherical vesicle [60]. For the same reasons REM does not impact the extracellular virions (mentioned in 3.6), the drug also cannot influence the viral adsorption stage.

### 3.8. Redox-Modulating Properties

The experimental results presented in Table 7 showed good FRAP and CUPRAC-reducing activities of COS, ranging in the millimolar range of Trolox equivalents. At this concentration, chitosan has the significant ability to chelate Fe II and can also quench DPPH free radicals and convert them to a colorless product, resulting in a decrease in absorbance at 517 nm.

Overproduction of redox oxidative stress (ROS) higher than the confident “critical” level can provoke genome instability and the activation of proliferation, in which normal cells begin to transform into malignant cells. For maintaining optimal cellular homeostasis, the regulation of cellular redox balance is crucial. When ROS generation is increased, it can initiate a cascade of the process—disruption of intracellular redox homeostasis and irreversible oxidative modifications of lipids, proteins, or DNA; this, in turn, may result in oxidative stress-induced cellular apoptosis.

The study of the redox-modulating capacity of drugs—removal of free radicals, chelation of metals, etc. contributes to the study of the pharmacological profile of newly synthesized components and to the clarification of the mechanisms of their toxicity. Investigation of their redox-modulating capacity, metal-reducing, and metal-chelating abilities, as well as their antiradical properties in the DPPH radical-containing system, are important aspects of their antioxidant activity.

The FRAP and CUPRAC methods are based on a single-electron transfer mechanism. FRAP assay is based on single electron transfer and measures the ability of an antioxidant to reduce ferric (FeIII) to ferrous (FeII) ions [60,61,62,63,64].

Our results showed some antioxidant activity in chitosan, but this completely disappeared upon incorporation into liposomes. This tendency is observed in the results of all conducted studies (Table 6). Therefore, in the liposomes, it is very likely that the binding of chitosan to the other components caused a decrease in its antioxidant capacity.

Moreover, the drugs that have the ability to chelate and reduce iron (III) ions are potential candidates for controlling ferroptosis and its destructive effects on healthy cells. Remdesivir has also shown an ability to chelate these ions; therefore, this property may complement its therapeutic effect during the course of infection.

## 4. Conclusions

The present study reports the design and characterization of composite liposomes suitable for encapsulation of the medicine Veklury^®^ (hydrophilic-modified drug SBECD–remdesivir). The liposomes were stabilized by the adsorption of chitosan or chitosan/hyaluronic acid film. Additionally, their surface was functionalized with the inclusion of an aptamer with specific affinity to the spike-protein of the coronavirus HCoV-OC43. The effect of the physicochemical characteristics of the polymers on the properties of the produced formulation was studied.

The loaded amount of the drug and the kinetics of release were estimated by UV–vis spectroscopy. The cytotoxicity of the studied formulations was determined against the HCT-8 cell line. A cytopathic effect inhibition test was used for the assessment of the antiviral activity of the compounds. The virucidal activity and the effect on the viral adsorption of the test samples were reported by the end-point dilution method, and the alteration in viral titer was determined as Δlgs compared to untreated controls.

The redox-modulating properties of the nanoparticles were studied in vitro in certain chemical model systems. Our investigations showed manifestation of promising tendencies for prolonged periods of the drug release and increased effectiveness of its antiviral action.

Among all studied versions of the delivery system, the most distinguished and suitable ones appear to include COS in their coatings, either as a single layer or as a part of a multi-layered film. First, the dimensional and stability properties of the formulations make them appropriate for lung delivery. Second, the oligosaccharide itself shows inhibition tendencies of the viral replication, virucidal activity, and a significant effect on reducing the viral adsorption on host cells.

## Figures and Tables

**Table 1:** Physicochemical characteristics of used polymers.

Polysaccharide	Mw, kDa (PDI *)	Degree Acetylation, %	Average Polymer Contour Length, nm
COS	5 (0.70)	<10	17
CS-L	50–190 (0.53)	15–25	453
CS-H	190–310 (0.84)	15–25	944
HA-L	8–15 (0.52)	-	9
HA-H	150–300 (0.60)	-	305

* Polydispersity index (PDI) was estimated by DLS measurements of pure polymer solution (0.1 mg/mL).

**Table 2:** Re-calculated encapsulation efficiency, EE%, loaded amount and capacity of liposomes with remdesivir stabilized by adsorption of chitosan layer in a diluted dispersion.

Chitosan Sample	Encapsulation Efficiency * EE, %	Loaded Amount, µg/mL	Loading Capacity ** LC, %
COS	99.4	299.4	61
CS-L	97.9	294.8	60
CS-H	97.8	294.7	60

* the error of the measurement is up to 10% [54]. ** the capacity is calculated taking into account the amount of capsules in dispersion (490 µg/mL).

**Table 3:** Reported structures for successful encapsulation of remdesivir.

Type of Carriers	Size nm	ζ-Potential mV	Encapsulation Efficiency EE, %	Loaded Amount µg/ml	References
Aerosolized nanoliposomal carrier	71.46 ± 1.35	−32.00 ± 2.00	99.79	2.5	[55]
Lipid nanocarriers	43.00 ± 0.60	−6.84 ± 0.59	99.94	499.8	[56]
Liposomes in aerosol	120	−7.00	62.41–83.93 depends on the drug/lipid ratio	N/xn	[22]
hyperbranched dendritic nanocarriers	185.0 ± 30.5	−7.00	14.10		[57]
Dendrimer-drug conjugates	1704–7172	1.14–23.70	37.97–48.43 depends on the conditions	339.0	[58]

**Table 4:** Cytotoxicity on HCT-8 cell lines and antiviral activity against human coronavirus (strain OC43): COS and HA-L are pure polysaccharide solutions, Liposomes-COS-A and Liposomes-COS-HA-A are samples of liposomes loaded with REM and aptamer A, stabilized by a monolayer of COS or bilayer of COS/hyaluronic acid (low molecular weight), respectively.

Sample	Cytotoxicity		Antiviral Activity	
	CC_50_ * Mean ± SD ** [µg/mL]	MTC *** [µg/mL]	IC_50_ * Mean ± SD ** [µg/mL]	SI ##
COS	812.6 ± 10.3	320.0	15.6	52.9
HA-L	˃1000.0	1000.0	110.0 ± 6.3	˃9.0
Liposomes-COS-A ^#^	149.7 ± 11.08	29.9	-	-
Liposomes-COS-HA-A ^#^	155.7 ± 10.18	29.9	-	-

* CC_50_—cytotoxic concentrations 50%; ** SD—standard deviation; *** MTC—maximum tolerable concentration; **##** SI—selectivity index, calculated from the ratio CC_50_/IC_50_. ^#^ relative to the concentration of a drug loaded in the liposomes (299.4 µg/mL).

**Table 5:** Virucidal activity of the samples of polysaccharide solution and liposomes against human coronavirus virions (strain OC-43): COS and HA-L are pure polysaccharide solutions, Liposomes-COS-A and Liposomes-COS-HA-A are samples of liposomes loaded with REM and aptamer A, stabilized by a monolayer of COS or bilayer of chitosan/hyaluronic acid (low molecular weight), respectively.

Sample	Δlg				
	15 min	30 min	60 min	90 min	120 min
COS	1.33	2.00	2.33	2.66	2.66
HA-L	2.00	2.00	2.50	2.75	3.00
Liposomes-COS-A	0.25	0.25	0.25	0.25	0.25
Liposomes-COS-HA-A	0.25	0.25	0.25	0.33	0.33

**Table 6:** Effect of the polysaccharides and liposomes on human coronavirus (strain OC-43) adsorption on cells (HCT-8).

Sample	Δlg				
	15 min	30 min	60 min	90 min	120 min
COS	1.75	1.75	2.0	2.5	3.25
HA-L	1.50	1.50	1.75	2.00	2.50
Liposomes-COS-A	0.25	0.33	0.33	0.50	0.50
Liposomes-COS-HA-A	0.33	0.33	0.33	0.50	0.50

**Table 7:** Redox-modulating properties of the produced structures.

Substance	Parameter			
	FRAP µM TEq/g	CUPRAC µM TEq/g	Fe II Chelation, %	DPPH Scavenging Effect, %
COS	0.0140 ± 0.002	6.52 ± 0.027	48.100 ± 11.400	16.400 ± 1.210
SBECD–REM (Vaklury^®^)	0.0016 ± 0.001	13.69 ± 1.020	-	-
Liposomes-COS-HA-A-drug	-	3.690 ± 0.820	-	-
